# The Gut Microbiota: A Potential Gateway to Improved Health Outcomes in Breast Cancer Treatment and Survivorship

**DOI:** 10.3390/ijms21239239

**Published:** 2020-12-03

**Authors:** Kara Sampsell, Desirée Hao, Raylene A. Reimer

**Affiliations:** 1 Faculty of Kinesiology, University of Calgary, 2500 University Drive NW, Calgary, AB T2N 1N4, Canada; kara.sampsell@ucalgary.ca; 2 Department of Medical Oncology, Tom Baker Cancer Centre and Cumming School of Medicine, 3330 Hospital Drive NW, Calgary, AB T2N 4N1, Canada; Desiree.Hao@albertahealthservices.ca; 3 Department of Biochemistry & Molecular Biology, Cumming School of Medicine, University of Calgary, 3330 Hospital Drive NW, Calgary, AB T2N 4N1, Canada

**Keywords:** gut microbiota, chemotherapy, breast cancer, cancer treatment, obesity, diet, prebiotics, probiotics, exercise

## Abstract

Breast cancer is the most frequently diagnosed cancer in women worldwide. The disease and its treatments exert profound effects on an individual’s physical and mental health. There are many factors that impact an individual’s risk of developing breast cancer, their response to treatments, and their risk of recurrence. The community of microorganisms inhabiting the gastrointestinal tract, the gut microbiota, affects human health through metabolic, neural, and endocrine signaling, and immune activity. It is through these mechanisms that the gut microbiota appears to influence breast cancer risk, response to treatment, and recurrence. A disrupted gut microbiota or state of ‘dysbiosis’ can contribute to a biological environment associated with higher risk for cancer development as well as contribute to negative treatment side-effects. Many cancer treatments have been shown to shift the gut microbiota toward dysbiosis; however, the microbiota can also be positively manipulated through diet, prebiotic and probiotic supplementation, and exercise. The objective of this review is to provide an overview of the current understanding of the relationship between the gut microbiota and breast cancer and to highlight potential strategies for modulation of the gut microbiota that could lead to improved clinical outcomes and overall health in this population.

## 1. Introduction

Within the past 15 years, research into the gut microbiota has increased at an exponential rate [1]. The gut microbiota refers to the resident and transient bacteria, viruses, fungi, protozoa, and archaea present in the human gastrointestinal tract [2]. To date, the vast majority of research on the gut microbiota has focused on bacteria since they comprise a significant component of the gut microbial community, numerous relationships to host health and disease have been established, and investigative methodologies have developed more quickly than for other abundant members of the gut microbiota, such as viruses [1,3]. While an optimal gut microbiota composition has not been identified, and may in fact not exist, a healthy host–microbiota balance involves communication via various metabolic and signaling pathways [1]. Disruptions to gut microbiota balance can occur and this ‘dysbiosis’ is implicated in a growing list of disease states such as obesity, diabetes, and various cancers [2]. The gut microbiota is one aspect alongside the host’s environmental exposures and epigenetic and genetic susceptibilities that can shape cancer risk [4]. The gut microbiota can potentially facilitate or impede carcinogenesis and may influence how an individual will respond to certain cancer therapies [4,5]. The mechanisms through which the microbiota might exert its influence on carcinogenesis and cancer treatments require further investigation; however, some relationships are postulated to exist via microbiota-derived metabolites, modulation of host metabolism, alteration of cytokine expression, and immune regulation [6,7,8,9].

As of 2018, cancer was the second leading cause of death worldwide and its incidence and mortality rates continue to increase globally in alignment with population growth and aging [10]. Breast cancer is the most prevalent cancer among women worldwide and carries the highest mortality rate [10]. In 2018, it was predicted that breast cancer would comprise nearly 1 in 4 (24.2%) new cancer diagnoses in women and it was the leading cause of female cancer death in 103 of 172 countries [10]. Considering the prevalence of cancer, and especially breast cancer, it has become increasingly important that scientists and clinicians understand the potential role that the microbiota plays in the development, progression, and treatment of the disease [5]. Harnessing this knowledge could lead to improved breast cancer prevention and enhanced treatment effectiveness. Cultivating a deeper understanding of the interactions between the gut microbiota and the host also has the potential to identify gut microbiota-targeted interventions [5]. The incidence of breast cancer and the number of survivors continues to grow, with many countries including the United States, Canada, England, Norway, Germany, Australia, and Japan reporting 85–90% five-year survival rates for women diagnosed between 2010 and 2014 [10,11]. This large and growing population will benefit from novel interventions to support health during treatment, in survivorship, and to prevent recurrence of the disease [12]. Although much remains to be learned about the microbiota–cancer relationship, the era of increasingly personalized medicine may see the status of an individuals’ gut microbiota emerge as a potentially useful characterization tool for predicting likelihood of specific treatment response, and with additional research, could serve as a possible interventional target to improve health outcomes in breast cancer [1,5,13,14].

Individuals with breast cancer face unique physical and psychological problems surrounding their diagnosis and treatment that can affect quality of life and clinical outcomes [15,16]. These include physical symptoms from the cancer itself, treatment side effects, depression, loss of lean body mass, fatigue, and anxiety [13,15,16,17,18,19]. These health concerns may compound on pre-existing conditions. In a cohort of Canadian women, 69–88% of individuals with breast cancer reported having one or more comorbidity at the time of diagnosis which is similar to the 73.8% who reported this in an American cohort [12,20]. Women who have had breast cancer also have a greater risk of developing an additional health condition such as obesity, cardiovascular disease, or mental illness [12,20]. These factors must be addressed in order to improve patient outcomes and quality of life [17,21]. Modulating the gut microbiota to improve host health may be a meaningful approach to this [22,23]. The aim of this review is to describe the understood relationship between the gut microbiota and breast cancer, particularly in the context of obesity, and identify potential interventional strategies and areas for further research that could improve health outcomes for this population.

## 2. Gut Microbiota in the Context of Breast Cancer and Dysbiosis

Numerous studies have suggested a role for breast and gut microbiota in the pathogenesis of breast cancer (reviewed in [24]). Microbial dysbiosis, or a disruption in the microbial community, has been observed in women diagnosed with breast cancer compared to healthy controls. For example, Xuan et al. showed that breast tumor tissue was enriched in *Methylobacterium radiotolerans* and that overall bacterial DNA load was reduced compared to paired healthy breast tissue [25]. Furthermore, Banerjee et al. showed that breast cancer subtypes have unique microbiota signatures with endocrine receptor (ER) and human epidermal growth factor receptor 2 (HER2) positive subtypes sharing similar microbial signatures compared to the distinct signature in triple-negative breast cancer tissues [26]. In terms of gut microbiota, Goedert et al. showed that postmenopausal women with breast cancer had lower alpha diversity and higher relative abundance of *Clostridiaceae*, *Faecalibacterium*, and *Ruminococcaceae*, alongside reduced abundance of *Dorea* and *Lachnospiraceae* compared to paired healthy controls [27]. In terms of microbial function, breast cancer in postmenopausal women has been associated with enrichment in gut microbial genes involved in lipopolysaccharide biosynthesis, iron complex transport system, vitamin B12 transport system, phosphotransferase (PTS) system, and secretion system, all of which have been associated with inflammatory conditions including breast cancer previously [28].

Although the defined composition of an ideal “healthy” microbiota remains elusive and in fact may not exist as a single entity, dysbiosis can increase risk for pathogenic infection, and is associated with inflammation and altered immune responses [4,5,29,30]. Dysbiosis commonly occurs through the loss of commensals, the proliferation of pathobionts (resident bacteria capable of causing disease), and/or a reduction in alpha diversity [31,32]. These shifts can occur following antibiotic treatment, chemotherapy, or radiation; all commonly utilized in cancer treatment [5,33,34,35]. Furthermore, dysbiosis has the potential to predispose an individual to infection from opportunistic pathogens capable of releasing toxins that can contribute to genomic instability and potentially, carcinogenesis [5,36]. In this dysbiotic state, the gut microbiota may lack sufficient diversity and resilience to prevent a bloom of bacteria such as certain strains of *Escherichia coli* which encode genes for toxins such as Colibactin or Cytotoxic Necrotizing Factor [36]. These group B2 and D *E. coli*-derived toxins are capable of altering the cell cycle, inducing DNA double strand breaks, and hijacking aspects of cell signaling which can contribute to genomic instabilities and abnormal cell activity in the intestinal tissues [36]. Dysbiosis is also associated with shifts in the metabolome (the metabolites in a given biological sample) toward an inflammatory state which is favorable for carcinogenesis [37].

The relationship between dysbiosis and inflammation may be bidirectional [37]. Although cancer itself is inflammatory, inflammation-inducing events such as cytotoxic chemotherapy or radiation disturb the gut microbiota, and these disturbances in turn are associated with inflammation through alterations in immune regulation, cytokine expression, and gut barrier function [37,38,39]. A healthy intestinal barrier consists of tightly packed epithelial cells with a thick mucus lining [37,39]. In a dysbiotic state, which can be caused by a chemical insult to the microbial community, the proteins (i.e., claudins, occludins, zona occludens) that maintain a tight junction between epithelial cells are compromised, in part due to increased claudin endocytosis [37,39]. Furthermore, dysbiosis could include a reduction in bacterial populations that contribute to maintenance of a thick mucus lining or that produce protective metabolites such as the immune-modulating short chain fatty acid butyrate. Collectively, these alterations can contribute to inflammation of the intestinal epithelium through increased barrier permeability and a decreased balance of colonic regulatory T cells to inflammation-associated Th17 cells [37,39,40]. Degradation of the mucosal lining and tight junctions between intestinal epithelial cells also allows bacterial particles such as lipopolysaccharide (LPS) to translocate into the blood stream, inciting inflammatory responses from the immune system on a systemic level [37]. Elevated circulating LPS has been associated with both liver and colorectal cancers [41,42]. Breast cancer metastasis has also been associated with increased circulating LPS through its ability to activate monocyte-mediated endothelial adhesion of circulating cancer cells [43]. In linking gut and breast microbiota, researchers have hypothesized that bacterial translocation from the gut to breast tissue is a possible mechanism through which distinct malignancy associated breast tissue microbiomes may develop [44]. The increase in gut permeability is often referred to as “leaky gut” and has also been implicated in several chronic inflammatory disease states such as obesity and irritable bowel syndrome as well as cognitive conditions such as depression and chronic fatigue [45]. Therefore, maintenance and restoration of gut barrier integrity in women with breast cancer during and after treatment may improve clinical outcomes.

The interactions between the host and the gut microbiota are highly complex which likely explains some of the variability in research findings that show the presence of certain bacterial species to be beneficial and in other cases detrimental. For example, *Akkermansia muciniphila* abundance was higher in individuals with colorectal cancer in a study done by Sheflin, but this same bacteria was key to the effectiveness of anti-PDL-1 immunotherapy in a study by Naito et al. [37,46]. These contradictory findings on *A. muciniphila* in relationship to cancer and treatment indicate the need for further research and underscore the likelihood that the desired abundance of key bacteria could be individual in nature based on the host, the pathology, and the treatments [4]. It is also important to consider how previous treatments and comorbid conditions may have affected an individual’s gut microbiota composition when considering what treatments will work best for them in the future since dysbiosis plays a potential role in the pathogenesis of cancer as well as in cancer therapy [4,5,37,47].

## 3. The Gut Microbiota and Breast Cancer Treatments

### 3.1. Chemotherapy

The gut microbiota can modulate host metabolism, inflammation, and immune responses; all crucial factors for tumorigenesis and dysregulated cell proliferation [48]. It is also through these physiological pathways that the gut microbiota has been shown to influence chemotherapy response and side effects [48]. The microbiota may affect treatment outcomes by metabolizing xenobiotic chemotherapy drugs, modulating immune response, or affecting local inflammation and gut barrier function directly or via its short chain fatty acid (SCFA) metabolites [48,49]. For example, in the nematode *Caenorhabditis elegans*, a bacterial diet of *E. coli* OP50 increased the efficacy of the chemotherapy drug 5-fluoro-2′-deoxyuridine whereas feeding *Comamonas* increased the efficacy of camptothecin [50]. Numerous nucleotide metabolism genes were identified in the bacteria that influenced drug efficacy in *C. elegans* [50]. The gut microbiota has also been linked to harsh side-effects of chemotherapy treatment. β-glucuronidases are enzymes encoded by both humans and microbes. The human encoded β-glucuronidase functions within lysosomes to breakdown structural glycosaminoglycans, while microbe-derived β-glucuronidases are expressed by species such as *Clostridium perfringens*, *Streptococcus agalactiae*, and *Bacteroides fragilis*, and have the capability to metabolize certain chemotherapy agents [51,52]. An example is Irinotecan, a chemotherapy agent commonly used to treat colon cancer and experimentally used to treat metastatic breast cancer, whose inactive metabolite can be reactivated by a β-glucuronidase present in the intestinal lumen, resulting in adverse drug effects such as severe diarrhea and intestinal damage [51,53,54,55]. β-glucuronidase-producing bacteria such as *Bacteroides* spp. and *Clostridium* spp. have been associated with accumulation of diarrhea-inducing metabolites from chemotherapy treatments such as Irinotecan and 5-Fluorouracil (5-FU) [53]. Diarrhea is a prevalent side-effect of several chemotherapeutic agents that is seen in 50–80% of patients and one that causes both discomfort and more severe complications such as dehydration [56,57]. *Bacteroides* spp. are common to the gut microbiota, and an increased abundance of *Clostridium* spp. is typical of dysbiosis following chemotherapy [48]. For example, the chemotherapy agent 5-FU was associated with post-treatment dysbiosis characterized by an increase of *Staphylococcus* and *Clostridium* spp. and a decrease in *Enterobacteriaceae*, *Lactobacillus*, and *Bacteroides* in the colon of rats treated with 5-FU compared to untreated controls [58]. Diarrhea in response to treatment with 5-FU in mice was found to be associated with increased expression of mRNAs encoding for inflammatory cytokines TNF-α, IL-1β, IL-6, Il-17A, and IL-22 as well as altered expression of intestinal and colonic aquaporins which are responsible for modulating fluid transfer through the gut barrier [59]. Inhibition of TNF-α did not affect the 5-FU-induced diarrhea, suggesting that the mechanism of the diarrhea may be independent from the associated upregulation of inflammatory cytokine expression [59].

Chemotherapy is also known to induce mucositis in many patients [60]. Gastrointestinal mucositis is an inflammatory condition that, like diarrhea, appears to develop in association with bacterial species shifts in the gut microbiota due to chemotherapy treatment [60,61]. For example, in mice treated with 5-FU, a decrease in the relative abundance of *Actinobacter* and an increase in *Verrucomicrobia* has been observed [60]. How long these bacterial shifts persist following treatment is not well understood. In a murine model of 5-FU-induced mucositis, levels of several chemokines and cytokines, including chemokine-1,-2, and -9, as well as Interleukin-4 were elevated [62]. Interleukin-4 can be proinflammatory and contributes to increased gut barrier permeability following 5-FU treatment, while chemokine-9 has been associated with gut epithelial damage due to its ability to phosphorylate p70 ribosomal S6 kinase which results in the inhibition of intestinal cell proliferation [8,62,63]. A murine model of 5-FU-induced mucositis was characterized by an elevation in several inflammation-associated cytokines at both the mRNA and protein level in serum and colon tissue alongside a decreased Firmicutes/Bacteroidetes ratio [62]. Additional phylum level shifts consisting of increased relative abundance of Verrucomicrobia, and decreased Proteobacteria and Cyanobacteria was observed [62]. Firmicutes and Bacteroidetes are the dominant phyla of the adult human gut microbiota [64]. In a heterogenous cohort of cancer patients, those classified as responders to their chemotherapy or immunotherapy treatments were found to have a significantly higher ratio of Firmicutes to Bacteroidetes after treatment compared to nonresponders, so this may be a potential metric of interest in cancer populations [14]. Nuclear factor kappa-light-chain-enhancer of activated B cells (NF-kB) and mitogen-activated protein kinase (MAPK) pathways can be activated by several inflammatory cytokines/chemokines which are elevated with 5-FU treatment, leading to dysregulated tight junction protein maintenance and promotion of a proinflammatory positive feedback signaling loop [62]. More work is needed to fully describe the mechanism by which gut microbiota shifts influence expression of these key signaling molecules that support gut epithelial injury from chemotherapeutics [62]. Abnormal cellular and molecular interactions due to gut microbiota may also play a role in chemotherapy-associated neuropathy. In a murine model comparing pain-sensitive B6 mice and pain resistant 129 mice, increased microglia presence in the spinal cord and brain stem was observed in the B6 mice compared to 129 mice in a microbiota-dependent fashion [19]. Significant negative associations were found between pain inhibition associated OTUs Porphyro_2 (*Porphyromonadaceae*) and Porphyro_16 and microglia presence in B6 mice but not in the 129 mice [19]. The researchers hypothesize that chemotherapy induces loss of gut barrier integrity which leads to systemic inflammation capable of driving this pain signaling [19]. With growing research on the mechanisms of these relationships, it may be possible in the future to target the gut microbiota to reduce an individual’s risk for adverse treatment effects.

It is also important to note that response to chemotherapy treatment can be affected by microbe-modulated immune responses and microbial translocation to lymphoid organs [48]. Platins, Cyclophosphamide, and 5-FU are chemotherapeutic agents with activity in breast cancer. Platinum-based chemotherapeutic drugs such as oxaliplatin and cisplatin have been shown to be ineffective in mice whose gut microbiota were depleted with antibiotic treatment as well as in germ free mice [48,65]. Disrupting the gut microbiota with antibiotics reduced the production of reactive oxygen species (ROS) by tumor-associated inflammatory cells, the production of which is required for oxaliplatin genotoxicity [65]. In a murine model, supplementation with *Lactobacillus acidophilus* alongside cisplatin offered a synergistic effect with the greatest survival and smaller tumor size compared to the groups treated with antibiotics and cisplatin as well as cisplatin alone [66]. Cyclophosphamide (CTX) effectiveness is similarly negatively associated with antibiotic administration [67,68]. Gut-resident *Enterococcus hirae* and *Barnesiella intestinihominis* have been reported as necessary for successful CTX response [69]. Both species support Th1 immune response [69]. *E. hirae* translocates from the gut to lymph nodes, increasing the CD8/Tregs ratio to support antitumor immunity while *Barnesiella intestinihominis* was found to promote infiltration of antitumor immune cells at the cancer site CTX [68,69]. Although the mechanism is undescribed, treatment with 5-FU has been found to be less effective in murine models of antibiotic-induced dysbiosis compared to controls [70]. Antibiotic-treated animals demonstrated an enrichment of the Proteobacteria species *Escherichia shigella* and *Enterobacter*, while the relative abundance of protective butyrate-producing bacteria including *Roseburia* decreased, which may be related to the subsequent poor 5-FU response [70]. Researchers note that 5-FU efficacy may be dependent on the pre-existing community of gut microbes and the mechanism may be related to the immune activation or xenobiotic metabolism capabilities of these microbes [70]. Although this effect was seen in the treatment of colorectal tumors with 5-FU, future studies should investigate the impact of dysbiosis on 5-FU treatment for other commonly treated tumor types, including breast tumors. Response to these chemotherapy agents is inhibited when the gut microbiota lacks species capable of supporting the necessary immune responses, and the treatments themselves are dysbiosis-inducing. At this time there are no published studies on the persistence (over a period of months) of dysbiosis following chemotherapy nor documentation of whether exercise or dietary interventions could resolve the dysbiosis and restore species that would benefit treatment effectiveness and tolerability. Additional clinical research is needed on the associations between gut microbiota and chemotherapy, but the composition of gut microbiota is being considered as a potential predictive biomarker for individual treatment response and target for improving outcomes [14,48,71].

### 3.2. Radiotherapy

Research addressing the impact of the gut microbiota on radiotherapy remains sparse; however, variability in gut microbiota has been reported as a potential contributor to the heterogeneity seen in tumor responses [48]. Radiotherapy is commonly used to treat breast cancer, so understanding potential influences on patient responses is clinically relevant. In one murine study investigating the relationship between the circadian rhythm and radiotherapy, researchers found that the mice with normal 12 h light/12 h dark schedules had improved survival and this was correlated with greater species richness in comparison to cohorts with altered light/dark schedules [72]. Radiotherapy may also induce dysbiosis which has been postulated to potentially relate to radiation toxicity [73]. In a murine model of severe radiotoxicity, fecal microbiota transplant (FMT) from control mice to irradiated animals significantly improved survival and mitigated toxicity [73]. Clinically, Ferreira et al. [74] reported that in individuals receiving pelvic radiotherapy, an association existed between gut microbiota composition and enteropathy, with an enrichment of *Clostridium*, *Roseburia*, and *Phascolarctobacterium* in those experiencing toxicities. It is important to consider that the radiation was localized closer to the gut microbiota for those individuals than it would be in an individual undergoing radiotherapy for breast cancer. These studies indicate the potential role for gut microbiota optimization during radiotherapy. More research is needed to elucidate the potential mechanisms and species-specific relationships present between gut microbes and radiotherapy response in various cancer types, including breast.

### 3.3. Immunotherapy

The use of immune checkpoint blockade agents represents an important advance in the treatment of cancer. Immune checkpoint inhibitors (ICIs) suppress the interaction of immune-response inhibiting receptors on T lymphocytes with their respective ligands which are found on the surface of cancer cells [75]. This interaction results in a greater T lymphocyte-mounted immune response to cancerous cells characterized by elevated CD4+ and CD8+ T cells in circulation and in the tumor microenvironment [75]. Recent preclinical studies and clinical trials support the use of anti-programmed cell death protein 1/programmed death ligand 1 pathway (anti-PD-1/PDL-1) therapy in some individuals with metastatic or triple negative breast cancer, which are difficult to treat [76,77]. Not all breast cancers are PD-1+, but therapies to disrupt this system has been reported as well tolerated for those individuals and it is being investigated as a potential adjuvant to conventional breast cancer therapies [76]. Clinical trial data suggests that the anti-PD-1/PDL-1 blockade drug atezolizumab may act synergistically alongside traditional chemotherapy with nab-paclitaxel, particularly for those with PD-1+ triple negative breast cancer [78]. Compared to the placebo–nab-Paclitaxel group, the Atezolizumab–nab-Paclitaxel group showed a significantly higher rate of progression-free survival at one year with 16.4% vs. 29.1% survival, respectively [78]. Multiple studies have demonstrated the necessity of the gut microbiota for response to the anti-PD-1/PDL-1 blockade [75,79]. The mechanism occurs at least partly through immune modulation [75]. T regulatory cells (Tregs) play a role in immune regulation and tolerance of self-antigens which are both key to anticancer immunity. Intratumoral Treg activity is measured via biomarkers such as the CD4/FOXP3 ratio, which was found to be elevated in mice who received an FMT from responder or nonresponder cancer patients and were subsequently cotreated with PD-1 and *Akkermansia muciniphila* [75]. In murine models, antibiotic treatment impedes response to the ICIs [5]. Primary resistance to this treatment is associated with dysbiosis and clinical outcomes are correlated with the relative abundance of *Akkermansia muciniphila*, whose presence was proven necessary for response to the therapy in a murine model [75]. *Bifidobacterium* spp. abundance, which is known to be a health-associated microbial group, has been linked to successful response to anti-PD-1/PDL-1 therapy [80]. In depleted mice, supplementation with *Bifidobacterium* spp. rescued a favorable anti-PD-1/PDL-1 response [80]. The mechanism of the relationship between gut microbiota and ICIs is not well understood but appears to occur partly through the recruitment of key immune cells to the tumor site [5,75,80]. A recently published study identified the gut microbe-derived metabolite inosine to play a key mechanistic role in immune cell activation at the tumor site during ICI treatment in murine models of colon cancer, bladder cancer, and melanoma [6]. Inosine is produced by bacteria such as *Bifidobacterium pseudolongum* and *A. muciniphila* in the upper gastrointestinal tract [6]. Monocolonization with *Bifidobacterium pseudolongum* in murine models of colon cancer prior to ICI treatment with anti-PDl-1 or anticytotoxic T-lymphocyte-associated protein 4 (CTLA-4), significantly increased antitumor immunity when compared to that of germ-free mice [6]. Elevated intratumoral IFN-γ+ CD4+ and IFN-γ+ CD8+ T cell infiltration was characteristic of increased immune checkpoint blockade efficacy with inosine administration [6]. *Lactobacillus johnsonii* and *Olsenella* species monocolonization also yielded significantly enhanced efficacy of ICIs, albeit to a lesser extent than *B. pseudolongum* monocolonization which aligns with the species’ ability to produce inosine [6]. Pretreatment optimization of the gut microbiota may be a strategy to improve response to ICIs; however, additional research is warranted on how to achieve that optimization as well as on the mechanism of microbial influence over ICI response. The influence of the gut microbiota should be considered as ICIs continue to be investigated for use in breast cancer treatment.

### 3.4. Hormone Therapies

Hormone receptor positive breast cancers are often treated with hormone therapies aimed at either lowering the amount of estrogen in the body or impairing the action of estrogen on breast cancer cells. Presently, no studies have investigated a link between response to hormone therapies and the gut microbiota, however this may be of interest considering the role of the gut microbiota in estrogen metabolism. The group of gut microbiome genes active in estrogen-related metabolism is termed the “estrobolome” and may serve as a useful biomarker [81,82]. Estrogen metabolized in the liver enters enterohepatic circulation which allows bacterial β-glucuronidase enzymes to deconjugate it to free estrogen that will enter systemic circulation [83]. β-glucuronidase producing bacteria belong to the genera *Clostridia* and *Ruminococcaceae* [83]. In addition to direct metabolism of estrogen, the gut microbiota can metabolize compounds known as phytoestrogens that are similar in structure and are capable of binding estrogen receptors [82]. This is true of microbiota derived metabolites of soy isoflavanoids and other plant lignans that act as phytoestrogens [82]. Higher circulating estrogen levels are linked to increased breast cancer risk in postmenopausal women [81]. Elevated deconjugation activity by the estrobolome can lead to excess reabsorption of deconjugated estrogen into circulation that would have otherwise been excreted, potentially increasing breast cancer risk [81]. However, phytoestrogenic compounds processed by the estrobolome have been known to regulate transcription factors to promote metabolism and clearance of carcinogens [82]. Although a recent meta-analysis showed that higher dietary intake of isoflavone (a phytoestrogen) was inversely associated with overall mortality and cancer recurrence in patients with breast cancer [84], the risk–benefit profile of phytoestrogens remains a topic of ongoing debate and has been reviewed in Senthilkumar et al. (2018) [85]. The relationship between the gut microbiota, estrogen, and breast cancer is complex and further investigation is needed, particularly with regards to the impact of the gut microbiota and estrobolome on hormone therapies for hormone receptor positive breast cancers.

## 4. Links between Obesity, Breast Cancer, and the Gut Microbiota

Obesity prevalence has risen dramatically in the past 30 years in North America and globally [86,87]. The U.S. is the epicenter of the obesity epidemic and saw a dramatic rise from about 15% of adults ages 20–74 having obesity in 1980 to 34% in 2008 [88]. Obesity is characterized by a body mass index (BMI) ≥ 30 and in addition to excess fat mass is often associated with chronic low-grade inflammation, insulin resistance, and impaired signaling of several hormones key to maintaining to metabolic health [89,90,91]. The presence of obesity increases the risk of developing breast cancer, breast cancer recurrence, and all-cause mortality for individuals with breast cancer [91,92]. Additionally, treatment efficacy is decreased in women with obesity undergoing systemic chemotherapy, and endocrine therapies prove to be significantly less effective than in nonobese women [91,92]. Individuals with obesity are also at higher risk for complications during surgery and radiation [92]. The mechanisms for these increased risks are complex. Adipose tissue is biologically active and capable of contributing to estrogen levels and producing inflammatory cytokines [91,92,93]. An excessive production of estrogen and inflammatory cytokines such as interleukin (IL)-1, IL-6, and tumor-necrosis factor (TNF) due to elevated fat mass in individuals with obesity may contribute to dysregulated biological processes that support carcinogenesis [93,94]. For example, TNF secretion from adipose tissue and circulating plasma IL-6, both of which play a role in insulin-response, were highly associated with obesity-associated insulin resistance in a human cohort [94]. Insulin resistance is recognized as a relevant risk factor for breast cancer [95]. Obesity causes increased circulating levels of insulin and insulin-like growth factors 1 and 2 which can bind insulin receptors, leading to downstream activation of RAS/MEK/MAPK and PI3K/Akt/mTOR pathways which upregulate S6K1, ultimately promoting protein and lipid biosynthesis supportive of dysregulated cell proliferation under conditions of excessive activation [93]. Low levels of adiponectin, a peptide involved in blood-glucose level modulation, are associated with obesity and have also been associated with incidence of breast cancer [96]. Researchers noted that low-adiponectin was also associated with significantly larger breast tumor sizes in individuals with obesity compared to those without obesity [96]. High estrogen and inflammation levels are also recognized risk factors for breast cancer recurrence [91]. In murine models of HER-2 positive breast cancer, obese animals show markedly faster tumor recurrence in comparison to nonobese controls, further indicating a link between the biological environment characteristic of obesity and increased breast cancer risk [91]. The notably detrimental impacts of obesity for breast cancer indicate a need to address or prevent the condition in those with active disease and survivors. A potential avenue for this lies in the relationship between the gut microbiota and obesity.

An obesity-associated gut microbiota differs significantly from a lean-associated gut microbiota [97,98,99]. In addition to lower bacterial diversity in those with obesity, elevated levels of *Lactobacillus* species and a relatively low abundance of *Bacteroides vulgatus*, *Bifidobacterium*, and *Akkermansia*, as well as a higher Firmicutes/Bacteroidetes ratio have been reported [97,98,100]. Fecal transplants from a mouse model of obesity into healthy germ-free mice resulted in the development of the obese phenotype in the previously healthy mice, demonstrating the existence of an obesity-associated gut microbiota [64]. An obesity-associated gut microbiota may harvest more energy from the diet in comparison to a lean-associated gut microbiota due to enrichment of genes encoding for enzymes capable of extracting energy from otherwise indigestible polysaccharides such as α- and β-galactosidases, and can modulate host genetics to promote greater deposition of lipids into adipose tissue, leading to weight gain [64]. More recent studies have demonstrated the ability of FMT from calorie-restricted mice as well as FMT from normal fat diet fed mice to attenuate characteristics such as weight gain and low leptin levels in recipient diet-induced obese mice, further emphasizing the influence of the gut microbiota in the context of obesity [101,102]. The influence of the gut microbiota on obesity and its associated metabolic characteristics has also been demonstrated using human fecal transplants. Fecal microbiota transplants from healthy individuals into those with obesity and metabolic syndrome increased insulin sensitivity over a 6-week follow-up period [103,104]. These studies showcase the influential role that members of the gut microbiota play in energy harvest, inflammation, and hormone signaling and highlight its potential as a target to improve the metabolic status of individuals with breast cancer to improve outcomes and reduce recurrence risk [105]. Diet and exercise are two areas often targeted in interventions to improve the health of individuals with obesity. In an observational study of women who had been treated for breast cancer, an associated 50% reduction in mortality risk was found for both nonobese and obese individuals who consumed at least five servings of fruits and vegetables per day and performed physical activity equivalent to six 30-min walks per week [106]. This study indicates the potential beneficial impact of incorporating healthy lifestyle choices, even if BMI remains in the obese range [106]. Additionally, diet and exercise are modifiable lifestyle factors that are investigated for their potential as interventions to address or prevent gut microbiota dysbiosis associated with obesity or cancer treatments.

## 5. Diet, Prebiotics, and Probiotics in Relationship to Breast Cancer

Overall diet composition, prebiotic intake, and probiotic intake are important factors that can shape an individual’s gut microbiota [107]. Prebiotics are substrates that are selectively utilized by host microorganisms and confer a health benefit, while probiotics are live microorganisms that, when administered in adequate amounts, confer a health benefit on the host [108,109]. An abundance of research has identified associations between dietary factors, the species present in the gut microbiota, and in some cases, disease states [107,110,111,112,113,114]. These associations may yield potential therapeutic avenues for dietary modifications and prebiotic or probiotic supplementation as complimentary to treatment and to support cancer prevention [107]. Fiber intake plays a notable role in shaping the gut microbiota [115]. The American Institute of Cancer Research endorses a diet high in fruits, vegetables, whole grains, and legumes and low in processed and red meats as cancer preventative [116]. In contrast, diet can also be cancer promoting through its modulation of the microbiota [117]. Certain microbes are known to cause direct inflammation of tissues or contribute to a carcinogenic metabolic environment [117]. For example, species of bacteria capable of fermenting protein, such as *Fusobacterium* genera, are known to be enriched in individuals with colorectal cancer compared to healthy cohorts [118]. Hydrogen sulfide resultant of microbial amino acid fermentation disrupts colonocyte barrier function [119]. Butyrate oxidation is necessary for ion absorption, mucin synthesis, membrane lipid synthesis, and detoxification processes in colonocytes and is dependent on an enzyme that is inhibited by hydrogen sulfide [119]. These processes support gut barrier function and normal intestinal epithelial cell proliferation and function [119]. As previously described, the gut barrier is integral to preventing endotoxemia and translocation of bacteria and their products from the intestinal lumen to distant locations in the body [39]. Loss of gut barrier integrity facilitates development of an inflammatory phenotype that is favorable for carcinogenesis [39,43,114]. This is not to say that individuals should not consume an adequate quantity of protein, but emphasis on other protective dietary factors is critical. Researchers have noted that inflammatory metabolic profiles associated with high proteolytic activity are mediated in individuals who consumed diets that were also high in fiber which is partially attributed to increased butyrogenesis [117,120].

Recent research demonstrated that prebiotic supplementation with inulin was associated with increased relative abundance of bifidobacteria and decreased levels of proteolytic metabolic products including ammonia and branched chain fatty acids in human fecal samples [121]. Consumption of fermented foods may also contribute to a protective metabolic environment due to their probiotic contents, notably *Lactobacillus casei* CRL431 [122,123,124,125]. In murine models of breast cancer, consumption of milk fermented with *Lactobacillus casei* CRL431 resulted in suppression of tumor angiogenesis and metastasis which was associated with decreased levels of proangiogenic factor IL-6, decreased infiltration of macrophages, and increased CD8+ and CD4+ lymphocyte response [122,123,124]. Adjuvant consumption of milk fermented with *L. casei* alongside administration of the chemotherapeutic Capecitabine was recently reported to decrease metastasis of breast cancer in mice, increase survival, decrease IL-6 levels, and mitigate common side effects such as weight loss, diarrhea, mucositis, and low red and white blood cell counts when compared to mice who consumed nonfermented milk alongside Capecitabine [125]. It would be interesting for future research to include gut microbiota and gut permeability analyses in the animals to further investigate the potential mechanisms of these findings. Overall, evidence is emerging that it is important for individuals at risk for breast cancer or fighting breast cancer to consume a diet that will help maintain a robust community of gut microbes that can inhibit an excess of inflammation-inducing microbes and molecules [37,107]. A primary goal should be to avoid or attenuate dysbiosis and metabolic conditions such as obesity which are associated with more favorable conditions for carcinogenesis and less favorable conditions for successful treatment response [37,68,70,75,91,93].

The gut microbiota profile can potentially play a protective role against cancer partly through its production of protective metabolites [126]. Butyrate is the primary protective SCFA and its intestinal concentration is dependent on both diet and the intestinal microbiota [126,127]. The chief short chain fatty acids produced by the gut microbiota include butyrate, propionate, and acetate which are metabolic products of microbial fermentation of dietary fiber [115]. Aside from serving as an energy source for colonocytes, butyrate can prevent histone de-acetylases from making epigenetic modifications that can lead to tumorigenesis [126,128]. Butyrate is also known to repress angiogenesis; therefore, slowing or inhibiting tumorigenesis [129]. Prominent butyrate producers include *Faecalibacterium prausnitzii*, *Roseburia intestinalis*, *Eubacterium rectale*, and *Roseburia* spp. [127,130]. Butyrate shows promise as an anticancer metabolite due to its anti-inflammatory properties, ability to induce cell differentiation and cancer cell apoptosis, and its protective histone hyper-acetylation activity [126,128,129,131]. The gut microbiota serves as the primary donor of acetyl in acetylation reactions that are responsible for regulating gene expression [126,132]. Therefore, facilitating butyrate production may be of interest in individuals with breast cancer or at risk for breast cancer. A diet high in fiber is considered cancer-protective partly for its facilitation of butyrate production and its favorability for proliferation of butyrate-producing microbes [107]. Biogenic amines are another group of metabolites that the gut microbiota produces via decarboxylation of dietary amino acids and that may influence host health [133]. These molecules can act as hormones, alkaloids, proteins, and nucleic acid precursors, as well as function in DNA stabilization [133]. Biogenic amines are also found in fermented food such as sauerkraut, cheese, and fish as a product of microbial fermentation of the amino acids present [134]. Cadaverine is a biogenic amine whose production appears to be repressed in individuals with early stage breast cancer [135]. Various members of the genera *Enterococcus*, *Enterobacter*, *Escherichia*, and *Proteus* have the ability to produce cadaverine [133]. In vitro, cadaverine supplementation at human serum reference levels decreased indicators of tumor aggression by suppressing cell shifts from epithelial to mesenchymal characteristics, cellular movement, and metastasis [135]. Eleven genes implicated in cell proliferation, movement, and adhesion were differentially expressed and largely suppressed with cadaverine treatment including MMP2, ERbb3, and Krt14, among others [135]. The cells treated with cadaverine demonstrated lower expression of genes implicated in movement. The biogenic amine exerts these effects through binding to trace amino acid receptors on target cells [135]. Lithocholic acid, a secondary bile acid and product of the gut microbiota, is found to exert similar effects, with a 10–20% decrease in breast cancer cell proliferation, decreased shift from epithelial to mesenchymal cell properties, and increased antitumor immune response in murine models and in vitro [136]. Lithocholic acid has also been found to induce cell death through increased p53 expression and can suppress dysregulated cell proliferation via a reduction in the expression of lipogenic metabolic targets such as SREBP-1 which are key to synthesis of molecules necessary for cell structures [137]. Levels of lithocholic acid and the gene of the enzyme necessary for its production were found to be low in the fecal DNA of women with early stage breast cancer compared to controls which indicates a lack of microbial production [137]. Promoting a gut microbiota that produces these protective metabolites in women with breast cancer could potentially improve their health outcomes. The mechanisms through which bacterial metabolites exert their effects are largely unknown and will require further investigation [133,135]. More research is needed in this area and on the outcomes of targeted dietary or supplementary interventions to increase microbial production of protective metabolic factors [133,135].

Prebiotics are substrates that are undigestible by the host and are often a type of fiber or polyphenol that serves as a nutrient that is selectively utilized by host microorganisms, eliciting health benefits [108]. Common prebiotics include fructooligosaccharide (FOS), inulin, and galactooligosaccharides (GOS) [108]. Although prebiotics are found naturally in foods such as asparagus, sugar beet, garlic, chicory, onion, Jerusalem artichoke, wheat, banana, and barley, the majority of research has administered FOS, GOS, inulin, and xylooligosacharides in higher doses in supplemental form [138]. These substrates have been found to increase abundance of *Lactobacillus* and *Bifidobacterium* among others [108,139]. In a murine model of obesity, oligofructose supplementation decreased circulating serum LPS levels by 40% over 12 weeks, thereby demonstrating its ability to influence systemic inflammation which can be incited by LPS in circulation [140,141]. Serum LPS has also been implicated in breast cancer metastasis, further highlighting the relevance of decreasing levels of serum LPS in circulation [43]. To date, no studies have focused on prebiotic supplementation alongside breast cancer therapy; however, prebiotics have been investigated in relationship to other tumor types. In a murine model, supplementation with inulin or mucin inhibited melanoma growth through distinct shifts in gut microbiota taxa that increased antitumor immune activity [142]. In addition, inulin was also found to limit growth of colon cancer tumors in a murine model [142]. *Akkermansia muciniphila* was most significantly enriched in the inulin-fed mice who experienced inhibited colon-cancer growth and *A. muciniphila* is also associated with treatment response in anti-PD-1/PDL-1 immunotherapy [75,142]. The mice in this study consumed a diet of 15% *w*/*w* inulin which is higher than that which can be achieved by humans; however, the associated gut microbiota shift and antitumor immune activation provide valuable mechanistic insights on the relationship between prebiotics, the gut microbiota, and tumor growth [142]. These findings indicate that further research on the benefit of prebiotics for other cancer types may be warranted. The ability of prebiotics to increase the abundance of taxa that benefit host health and decrease inflammation levels could be helpful to individuals with breast cancer before and during treatment to promote health, and after treatment to attenuate dysbiosis; however, more research is needed on this topic.

Probiotics have been extensively studied for their potential as a safe and effective means of providing beneficial microbiota to a host [107]. The latest consensus statement defines probiotics as live microorganisms that, when administered in adequate amounts, confer a health benefit on the host [109]. As the authors of the consensus statement point out, there are many products available to consumers that are called probiotic but “too often they do not meet minimum criteria, such as defined contents, appropriate viable count at end of shelf-life and suitable evidence for health benefit” [109]. Therefore, rigorous scientific evidence is needed to meet the definition of probiotic. Specific to cancer, it has been observed that individuals with colorectal cancer undergoing 5-FU chemotherapy who were administered probiotic *Lactobacillus rhamnosus* GG experienced less abdominal discomfort and diarrhea [143]. Similar effects may be possible for individuals with breast cancer undergoing chemotherapy but have not yet been shown. Although probiotics are generally considered safe, septicemia has been observed following *Bacillus subtilis* supplementation in a severely immunocompromised individual and evidence of bacterial translocation from the gut from the blood has also been noted in critically ill patients who were administered *Lactobacillus rhamnosus* GG [144,145]. Due to this risk, it is important to weigh the benefits and risks of administering probiotics on an individual basis. Despite this, there are several potential benefits of probiotic administration that are worth discussing. In a murine model of breast cancer, administration of *Lactobacillus acidophilus* ATCC4356 strain induced production of immunomodulatory IL-2 and was associated with decreased tumor growth rates [146]. In another instance of *L. acidophilus* supplementation alongside cisplatin, a common chemotherapy agent, the combination led to smaller tumor size and greater survival in a murine model when compared to cisplatin treatment alone [66]. While antibiotic-treated mice had diminished levels of key immune markers, those coadministered cisplatin and *L. acidophilus* exhibited greater expression of IFN-γ, GZMB, and PRF1, demonstrative of an enhanced antitumor immune response [66]. In vitro, researchers have found that *L. acidophilus*, *L. gasseri*, *L. fermentum*, and *L. rhamnosus* promote the gut barrier integrity by upregulating expression of E-cadherin, an adherence junction protein [147]. *Lactobacillus rhamnosus* GG (LGG) is the most widely studied probiotic in relationship to cancer [5]. LGG is well known for its anti-inflammatory properties [5]. Its presence shifts gene expression in gut epithelial cells to support an anti-inflammatory profile demonstrated by an association with downregulation of proinflammatory CXCL-2, IL-6, and IL-8, among others [5,148]. In a murine model LGG administration attenuated intestinal epithelial damage and inflammation as a result of 5-FU chemotherapy, preserving microbiota balance, and maintaining gut barrier integrity [149]. These effects have been seen both in vitro and in vivo and two clinical studies are currently focused on the effect of daily LGG intake on patients undergoing cytotoxic therapies for cancer [5]. The abundance and strength of the research on LGG makes it the most viable current option for a probiotic as a complementary therapy during cancer treatment [5]. Knowing this, it seems possible that other probiotics may have similar beneficial effects for individuals with cancer; however, more preclinical and clinical research is needed on which strains are beneficial during specific treatments before probiotic administration can be considered both safe and customizable for all individuals.

## 6. Exercise and the Gut Microbiota

While the impact of diet on gut microbiota is supported by a robust body of research, the impact of exercise has more recently become a factor of interest. It has been demonstrated that exercise can alter the gut microbiota independently from diet [150]. Support for exercise as a beneficial modifier of the gut microbiota originated from observational studies that show that a greater ratio of Firmicutes to Bacteroidetes is correlated with higher VO_2_ max and that women who performed at least 3 h of exercise per week had higher abundance of several butyrate-producing bacteria as well as *Akkermansia muciniphila* which has been associated with lean body mass index [150,151]. Although these studies show positive correlations between beneficial microbiota and exercise, they were done in healthy individuals and failed to control for other factors such as diet which are known to affect the gut microbiota, indicating that the results may not be attributable to exercise alone [150,151]. Additionally, because these studies were cross-sectional, it is difficult to determine whether the microbiota profile or the higher VO_2_ max was present first [150,151]. More recent studies have been performed longitudinally in a controlled setting and demonstrate that 30–60 min of aerobic exercise performed three times per week is enough to induce significant changes in gut microbiota, although the changes differ in lean individuals compared to individuals with obesity [150]. For example, *Faecalibacterium* species increased in lean subjects but decreased in subjects with obesity and *Bacteroides* species decreased in the lean subjects and increased in the subjects with obesity [150]. It is also important to note that 6 weeks of sedentary behavior following the exercise intervention reversed any changes that were seen in the gut microbiota during the exercise intervention, indicating that the effects of exercise are transient and easily reversed [150]. In premenopausal women, those meeting the World Health Organization’s recommendation for 150 min of moderate aerobic activity each week presented greater abundances of *Akkermansia muciniphila*, *Faecalibacterium prausnitzii*, and *Roseburia hominis* compared to those who were sedentary [152]. These species have demonstrated health-promoting effects such as maintenance of the gut barrier [152]. *Akkermansia muciniphila* is often enriched in the gut microbiota of athletes which suggests that its presence could be promoted by physical activity [153]. Investigating the effect of exercise on the gut microbiota is difficult due to the additional lifestyle factors that may shape the gut microbiota, especially diet. Studies with strictly controlled diet would enhance researchers’ ability to investigate potentially causal relationships between exercise and changes in the gut microbiota. There are currently no studies on the effects of exercise on the gut microbiota of individuals with cancer. More research is needed on the effects of different exercise types and intensities, exercise in combination with prebiotics or probiotics, and exercise in a variety of populations [153,154].

It is important to note the additional benefits of exercise for women with breast cancer alongside its ability to beneficially manipulate the gut microbiota. A sedentary lifestyle has been associated with cancer and many other chronic diseases [152]. There is strong evidence that being physically active decreases the risk of breast cancer in pre- and postmenopausal women [155]. Exercise has been proven as a safe intervention for cancer patients to improve their fatigue, physical function, and quality of life [156]. The current American College of Sports Medicine guidelines for exercise in cancer populations states that exercise should play a key role in the prevention and control of cancer [157]. In murine models of breast cancer, exercise increases sensitivity to chemotherapeutics through decreased hypoxia and regulation of vascularity, leading to improved outcomes via direct tumor suppression [158]. Additionally, exercise slows the growth of certain tumor types through its ability to induce vascular normalization and increase immune activity at the tumor site in murine models [156]. Exercise can also improve several chemotherapy side-effects such as nausea and vomiting [21]. Observational data demonstrates a linkage between self-reported exercise and reduced rates of development and recurrence of several cancers [156]. A prospective study of women with breast cancer found that meeting the minimum guidelines for physical activity both before and after diagnosis is associated with significantly reduced risk of recurrence or mortality [159]. Interestingly, those who performed comparatively lower levels of activity prediagnosis, during treatment, and postdiagnosis experienced similar benefits to those who performed higher levels of activity at each time point [159]. This indicates that even smaller amounts of regular physical activity yield significant benefits for this population [159]. It is also important to note that those who did not meet guidelines prediagnosis but met them 2 years postdiagnosis still experienced a 46% decreased risk of recurrence and 43% decreased risk of mortality [159].

These beneficial association can be explained by exercise’s ability to improve body composition, decrease sex hormone bioavailability, improve insulin sensitivity, decrease levels of inflammatory biomarkers, and promote DNA repair [159]. Key growth and energy metabolism pathways including mTOR and AMPK are differentially regulated during exercise which could impact tumor growth, though researchers note that the impact is not yet mechanistically understood [160]. High levels of intratumoral lactate inhibits the infiltration of natural killer cells and T-lymphocytes; however, exercise has been shown to lower intratumoral lactate levels which could maximize antitumor immune potential [160]. This is of interest given the Warburg effect where proliferating tumor cells consume glucose at a high rate and release lactate [161]. Decreased tumor lactate concentrations following endurance exercise have been associated with a reduction in monocarboxylate transporter-1 and lactate dehydrogenase-A expression and increased expression of lactate dehydrogenase-B [162]. In a murine model, the exercised animals demonstrated enrichment of *Faecalibacterium prausnitzi* and decreased expression of the inflammatory enzyme cyclooxygenase-2 (COX-2) in the proximal and distal gut epithelium which indicates improved gut barrier integrity and is hypothesized to be resultant of increased butyrate production [163]. In addition to the above benefits, resistance exercise has been shown to increase lean body mass [164]. According to a meta-analysis, 27.7% of individuals with cancer have low muscle area based on computed tomography tests, and this is associated with poor survival rates [164]. Therefore, it is important to promote and maintain lean body mass in individuals with breast cancer, and exercise may address this. It is possible that these benefits are also related to change in the gut microbiota, but research is needed to explore these areas.

Exercise has been associated with improved psychosocial outcomes in individuals with cancer [165]. Greater levels of physical activity correlated with improved health-related quality of life [165]. The mechanism by which this correlation exists has not been elucidated, but one potential avenue of interest that requires additional investigation is possible modulation of the gut–brain axis [166]. The gut–brain axis is a relatively novel paradigm that seeks to characterize the way that the gut microbiota and brain communicate bidirectionally [167]. The presence of mood-related hormones in the gut contributed to recent research to explore the gut–brain axis [167]. Serotonin is a primary mood and cognition regulating hormone that plays a role in both gut and brain function and 90–95% of an individual’s serotonin is found in the gut [168]. Levels of blood serotonin have been noted to change in response to exercise and are associated with decreased depressive symptoms compared to nonexercised controls [169]. Many gut microbes have been reported to produce and/or consume neurotransmitters including GABA, norepinephrine, dopamine, serotonin, acetylcholine, and histamine [168]. Currently, no data exists on whether exercise is associated with shifts in abundance of these species. However, these gut-microbiota derived neurotransmitters are postulated to act on the gut–brain axis [170]. Increases in Firmicutes, SCFA production, and overall species diversity have been associated with exercise [171]. These changes are in turn were correlated with decreased anxiety and depression [171]. Additional research is needed to establish a direct relationship between those factors. In a pilot study investigating the effect of exercise in breast cancer survivors, correlations between gut microbiota beta diversity and fatigue and depression were seen as well as between gut microbiota composition and cardio-respiratory fitness [166]. These findings call for further research investigating the influence of exercise in cancer populations and the correlations between gut microbiota and psychosocial outcomes [166]. Focus should also be placed on investigating the mechanisms through which a potential relationship between exercise and the gut–brain axis may exist.

## 7. Conclusions

**Figure 1:**
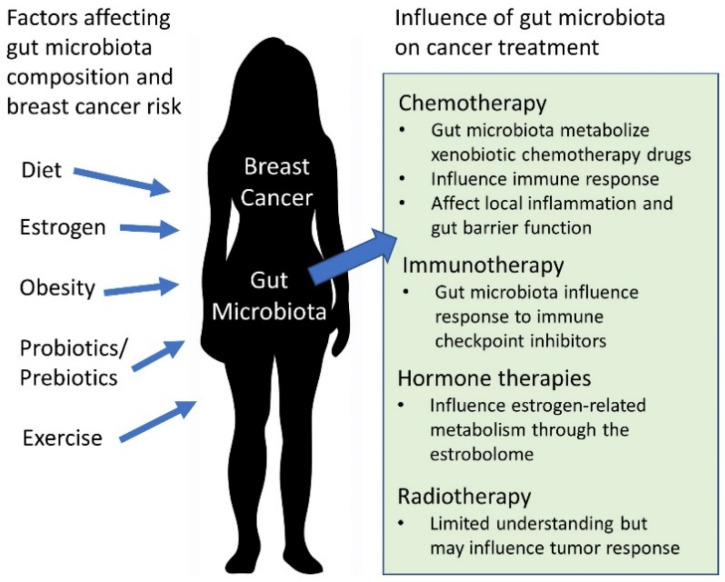
Potential relationship between gut microbiota and breast cancer.

The gut microbiota’s relationship with host physiology provides a potential avenue for it to influence the development, progression, and treatment of breast cancer. The gut microbiota presents as a possible novel target to improve treatment efficacy and long-term health outcomes by beneficially shaping host metabolism, molecular signaling, and immune responses (Figure 1).

Additional research is needed to elucidate the mechanisms through which potential associations exist between the gut microbiota and response to treatments for breast cancer. Additionally, further characterization of the gut microbiota associated with response to these treatments is necessary and could potentially translate into strategies to optimize the gut microbiota prior to treatment or development of predictive models. Modulation of the gut microbiota has the potential to decrease several common side effects of breast cancer treatment and make effective treatments more tolerable. Diet, prebiotic and probiotic supplementation, and exercise show promising signs as strategies to optimize the gut microbiota pretreatment and attenuate treatment or disease-associated dysbiosis to promote health and reduce recurrence risk. These interventional strategies may also improve overall metabolic health and reduce the negative impact of common comorbidities such as obesity. Additional animal and clinical studies are needed to identify safe and effective ways to incorporate these strategies into clinical treatment pathways. Strengthening the current understanding of the interactions between the gut microbiota, breast cancer risk, and breast cancer treatments could lead to safe and effective gut microbiota-based interventions that will improve health outcomes in this population.

## Abbreviations

COX-2	Cyclooxygenase-2
CTX	Cyclophosphamide
FMT	Fecal microbiota transplant
FOS	Fructooligosaccharides
5-FU	5-Fluorouracil
GOS	Galactooligosaccharides
GZMB	Granzyme b protein
ICI	Immune checkpoint inhibitors
IFN-γ	Interferon gamma
IL-6	Interleukin-6
LPS	Lipopolysaccharide
PRF1	Perforin-1
PTS	Phosphotransferase system
ROS	Reactive oxygen species
SCFA	Short-chain fatty acids
SREBP	Sterol-regulatory element binding protein
TNF	Tumor necrosis factor
